# Efficacy and Safety of Alirocumab in Patients with Heterozygous Familial Hypercholesterolemia and LDL-C of 160 mg/dl or Higher

**DOI:** 10.1007/s10557-016-6685-y

**Published:** 2016-09-13

**Authors:** Henry N. Ginsberg, Daniel J. Rader, Frederick J. Raal, John R. Guyton, Marie T. Baccara-Dinet, Christelle Lorenzato, Robert Pordy, Erik Stroes

**Affiliations:** 1 Columbia University College of Physicians and Surgeons, Irving Institute for Clinical and Translational Research, Columbia University, 622 West 168th Street, New York, NY 10032 USA; 2 Departments of Medicine and Genetics, Perelman School of Medicine of the University of Pennsylvania, Philadelphia, PA USA; 3 Faculty of Health Sciences, University of Witwatersrand, Johannesburg, South Africa; 4 Duke University Medical Center, Durham, NC USA; 5 Sanofi, Montpellier, France; 6 Sanofi, Paris, France; 7 Regeneron Pharmaceuticals, Inc., Tarrytown, NY USA; 8 Department of Vascular Medicine, Academic Medical Center, University of Amsterdam, Amsterdam, Netherlands

**Keywords:** Alirocumab, Cardiovascular disease prevention, Cholesterol-lowering drugs, Familial hypercholesterolemia, LDL-C, PCSK9

## Abstract

**Purpose:**

Even with statins and other lipid-lowering therapy (LLT), many patients with heterozygous familial hypercholesterolemia (heFH) continue to have elevated low-density lipoprotein cholesterol (LDL-C) levels. ODYSSEY HIGH FH (NCT01617655) assessed the efficacy and safety of alirocumab, a proprotein convertase subtilisin/kexin type 9 monoclonal antibody, versus placebo in patients with heFH and LDL-C ≥ 160 mg/dl despite maximally tolerated statin ± other LLT.

**Methods:**

Patients were randomized to subcutaneous alirocumab 150 mg or placebo every 2 weeks (Q2W) for 78 weeks. The primary endpoint was percent change in LDL-C from baseline to week 24.

**Results:**

Mean baseline LDL-C levels were 196.3 mg/dl in the alirocumab (*n* = 71) and 201.0 mg/dl in the placebo groups (*n* = 35). Significant mean (standard error [SE]) reductions in LDL-C from baseline to week 24 were observed with alirocumab (−45.7 [3.5] %) versus placebo (−6.6 [4.9] %), a difference of −39.1 (6.0) % (*P* < 0.0001). Absolute mean (SE) LDL-C levels were reduced from baseline by 90.8 (6.7) mg/dl with alirocumab at week 24, with reductions maintained to week 78. Treatment-emergent adverse events were generally comparable between groups. Injection-site reactions were more frequent in the alirocumab group (8.3 %) versus placebo (5.7 %); most were mild in severity and did not result in study medication discontinuation.

**Conclusions:**

In patients with heFH and very high LDL-C baseline levels despite maximally tolerated statin ± other LLT, alirocumab 150 mg Q2W demonstrated significant reductions in LDL-C levels with 41 % of patients achieving predefined LDL-C goals. Alirocumab was generally well tolerated.

**Electronic supplementary material:**

The online version of this article (doi:10.1007/s10557-016-6685-y) contains supplementary material, which is available to authorized users.

## Introduction

Heterozygous familial hypercholesterolemia (heFH) is associated with high levels of low-density lipoprotein cholesterol (LDL-C), which predisposes to premature atherosclerosis and cardiovascular disease [1, 2]. LDL-C levels can be in the range of ~200–400 mg/dl (5–10 mmol/L) if untreated and are >160 mg/dl (4.14 mmol/L) in a number of affected adults, despite treatment with available lipid-lowering therapies (LLTs) [3, 4]. Due to lifelong exposure to elevated LDL-C plasma levels, patients with heFH are characterized by a markedly increased risk of coronary heart disease compared with the general population [1].

Current LDL-C-lowering treatments include statins and ezetimibe, which have both been shown to contribute to a reduction of coronary mortality [5, 6]. Other LLTs, such as bile acid sequestrants and fibrates, may also be added to statin therapy to further reduce LDL-C levels [7]. However, despite treatment with the current standard of care, many patients with heFH struggle to achieve sufficient LDL-C reductions, with previous research reporting that only ~20 % of patients with heFH achieved a pre-defined LDL-C target level of <100 mg/dl with existing cholesterol-lowering treatment options [7, 8]**.**

Proprotein convertase subtilisin/kexin type 9 (PCSK9) targets the degradation of hepatic LDL receptors, thereby reducing the ability of the liver to remove LDL-C from the circulation [9]. Inhibitors of PCSK9 are now available in the United States and Europe to reduce LDL-C in patients with heFH, atherosclerotic cardiovascular disease, and/or cardiovascular risk factors [10–13]. In phase 1 studies, usually conducted in a limited number of highly selected investigator sites, PCSK9 inhibitors have been shown to reduce LDL-C levels in patients with heFH [14, 15]**.** Results from phase 2 and 3 studies to date with the fully human monoclonal PCSK9 antibody alirocumab have demonstrated considerable potential for the reduction of LDL-C levels in different patient populations [11, 16–25], with significant reductions in mean LDL-C levels of 68 % observed in a phase 2 study that included patients with heFH (placebo: 11 %) [11]. In the phase 3 ODYSSEY program, a large proportion of patients with heFH were included (>1200 patients); in three studies published to date, alirocumab demonstrated significant LDL-lowering in patients with heFH [19, 25].

The phase 3 ODYSSEY HIGH FH study was a placebo-controlled study evaluating the lipid-lowering efficacy and safety of alirocumab 150 mg every 2 weeks (Q2W) as add-on therapy in patients with heFH and baseline levels of LDL-C ≥ 160 mg/dl despite maximally tolerated statin therapy (with or without other LLTs). The results of ODYSSEY HIGH FH are presented here.

## Methods

The ODYSSEY HIGH FH study was a multicenter, randomized, double-blind, placebo-controlled, phase 3 trial (ClinicalTrials.gov identifier: NCT01617655). It was conducted at 33 sites across Canada, the United States, the Netherlands, Russia, and South Africa from June 2012 to January 2015. The study was performed in accordance with the ethical principles that have their origin in the Declaration of Helsinki and all applicable amendments laid down by the World Medical Assemblies and the International Conference Harmonization guidelines for Good Clinical Practice. The protocol was approved by the local institutional review board and independent ethics committee at each site, and all patients provided written informed consent prior to participation.

### Patients

Full details of the inclusion and exclusion criteria for the ODYSSEY HIGH FH trial have been published previously [10]. Briefly, patients with heFH and LDL-C ≥ 160 mg/dl on a maximally tolerated stable daily dose of statin, with or without other LLT, for at least 4 weeks (6 weeks for fenofibrate) prior to the screening visit were included.

The diagnosis of heFH was made by either genotyping (17.8 %) or clinical criteria (82.2 %) (Table 1). For those patients not genotyped, the clinical diagnosis was based on either Simon Broome criteria for definite FH [26], or the World Health Organization/Dutch Lipid Network criteria (score > 8 points) [27].

**Table 1:** Baseline characteristics (all randomized patients)

All patients on background of maximally tolerated statin ± other lipid-lowering therapy	Alirocumab 150 mg Q2W (*n* = 72)	Placebo (*n* = 35)
Baseline demographics		
Age, years, mean (SD)	49.8 (14.2)	52.1 (11.2)
Male, % (n)	48.6 (35)	62.9 (22)
Race, White, % (n)	88.9 (64)	85.7 (30)
BMI, kg/m^2^, mean (SD)	28.8 (5.2)	28.9 (4.2)
Confirmation of heFH diagnosis by genotyping, % (n)	19.4 (14)	14.3 (5)
Confirmation of heFH diagnosis by WHO/Simon Broome criteria, % (n)	80.6 (58)	85.7 (30)
CHD history, % (n)	43.1 (31)	62.9 (22)
CHD risk equivalent,^a^ % (n)	18.1 (13)	14.3 (5)
Hypertension, % (n)	55.6 (40)	60.0 (21)
Type II diabetes, % (n)	12.5 (9)	17.1 (6)
Current smoker, % (n)	16.7 (12)	25.7 (9)
Lipid medication		
Statin use, % (n)	100 (72)	100 (35)
High-intensity statin use,^b^ % (n)	73.6 (53)	71.4 (25)
Simvastatin 80 mg, % (n)	5.6 (4)	8.6 (3)
Other LLT use, % (n)	22.2 (16)	37.1 (13)
Ezetimibe use, % (n)	19.4 (14)	34.3 (12)
Baseline lipid parameters, mg/dl		
LDL-C (calculated), mean ± SD	196.3 ± 57.9^c^	201.0 ± 43.4
Non-HDL-C, mean ± SD	223.9 ± 58.8	231.5 ± 47.6
Total cholesterol, mean ± SD	273.5 ± 57.5	276.4 ± 46.8
ApoB, mean ± SD	138.2 ± 32.0^c^	146.6 ± 28.3^d^
Lp(a), median (Q1:Q3)	22.0 (8.0:50.0)^c^	30.0 (11.0:42.0)^d^
HDL-C, mean ± SD	49.6 ± 14.0	44.9 ± 11.3
Fasting TGs, median (Q1:Q3)	131.5 (87.5:160.5)	122.0 (95.0:193.0)

*Apo* apolipoprotein, *BMI* body mass index, *CHD* coronary heart disease, *HDL-C* high-density lipoprotein cholesterol, *heFH* heterozygous familial hypercholesterolemia, *LDL-C* low-density lipoprotein cholesterol, *LLT* lipid-lowering therapy, *Lp(a)*, lipoprotein(a), *Q2W* every 2 weeks, *SD* standard deviation, *TGs* triglycerides, *WHO* World Health Organization

^a^CHD risk equivalents were defined as ischemic stroke, peripheral arterial disease, moderate chronic kidney disease, and diabetes mellitus (only if two or more risk factors present)

^b^High-intensity statin therapy was defined as atorvastatin 40–80 mg daily or rosuvastatin 20–40 mg daily

^c^ *n* = 71

^d^ *n* = 34

Maximally tolerated statin therapy included daily doses of simvastatin (80 mg, if on this dose for >1 year), atorvastatin (40–80 mg), or rosuvastatin (20–40 mg). Patients not on any of these statin doses were treated with the dose of daily atorvastatin, rosuvastatin, or simvastatin which was considered appropriate for the patient, according to the investigator’s judgment. Additional exclusion criteria were fibrates (other than fenofibrate), fasting serum triglycerides >400 mg/dl (4.52 mmol/L) and known history of homozygous FH.

### Study Design

Following a screening period of up to 3 weeks, all eligible patients were randomized 2:1 to alirocumab 150 mg Q2W administered subcutaneously via 1 ml prefilled pen, or matching placebo, for 78 weeks (Supplementary Fig. 1). Randomization was stratified according to history of myocardial infarction or ischemic stroke, as well as intensity of statin treatment (atorvastatin 40–80 mg daily, rosuvastatin 20–40 mg daily vs. simvastatin, atorvastatin <40 mg daily, or rosuvastatin <20 mg daily). At the end of the 78-week double-blind treatment period, patients could enter an open-label extension study, during which they all received alirocumab 150 mg Q2W. Those patients opting out of the open-label treatment period underwent an 8-week follow-up. Patients were asked to adhere to a stable diet in line with the National Cholesterol Education Program Adult Treatment Panel III Therapeutic Lifestyle Changes diet or equivalent, and received statin therapy at the maximally tolerated daily dose with or without other LLT throughout the study.

**Fig. 1:**
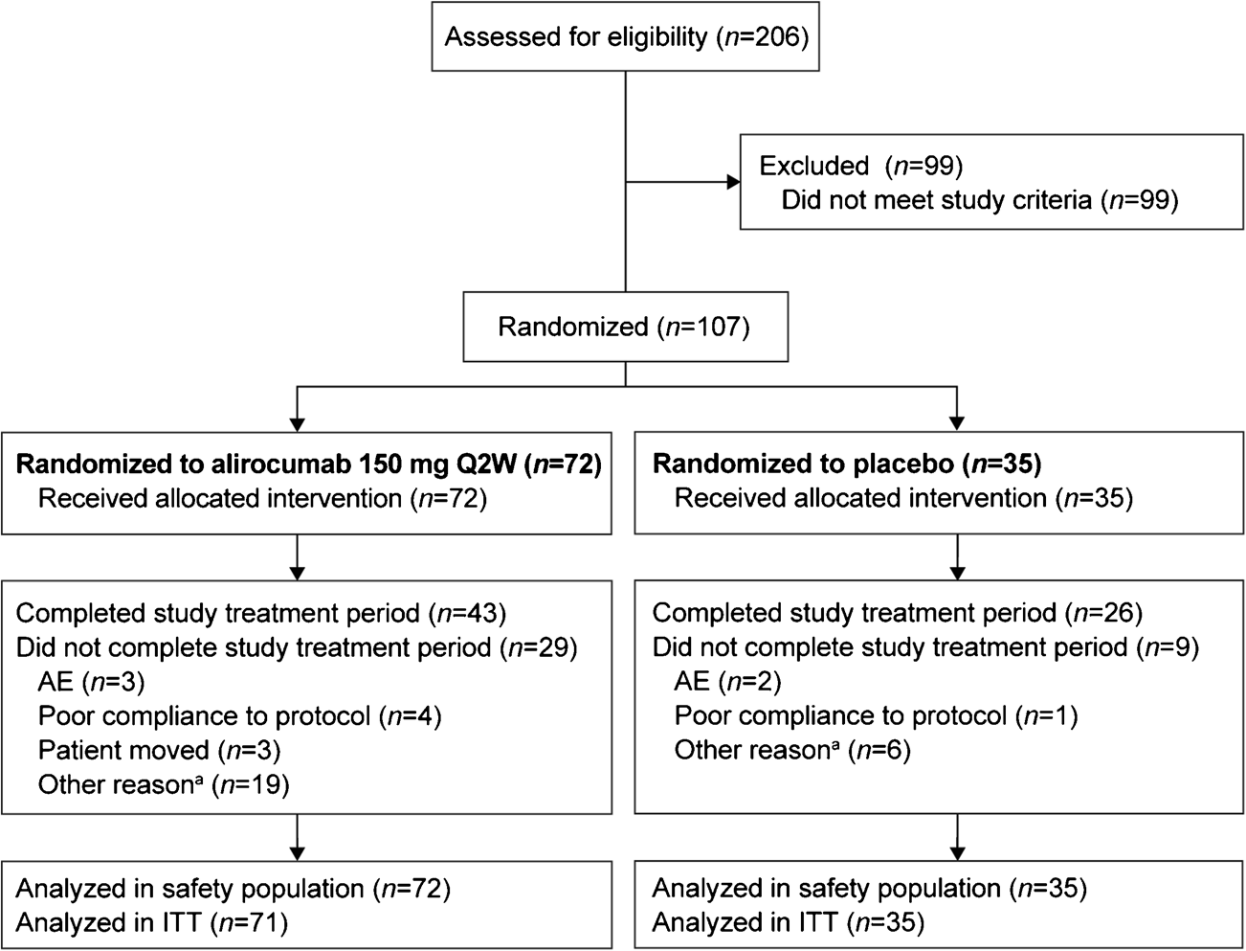
Patient flow through the study. ^a^Includes 14 patients (alirocumab *n* = 12; placebo *n* = 2) who completed the 18 months double-blind treatment period (at least 76 weeks of exposure and visit week 78 performed) but did not meet the definition of “completer per CRF”, eight patients with site closure/site location unavailable (alirocumab *n* = 5; placebo *n* = 3), three alirocumab-treated patients with other reasons (not willing to continue the study [no specific reason provided] *n* = 1; new job too far from research unit *n* = 1; withdrawal due to cholesterol results done independently *n* = 1). All patients on background of maximally tolerated statin ± other lipid-lowering therapy AE, adverse event; CRF, case report form; ITT, intent-to-treat; Q2W, every 2 weeks

### Endpoints and Assessments

The primary efficacy endpoint was the percent change in calculated LDL-C from baseline to week 24 analyzed with the use of an intent-to-treat (ITT) approach. Secondary efficacy endpoints included LDL-C percent change from baseline to week 24 (on-treatment analysis) and from baseline to weeks 12, 52, and 78 (ITT); percent change in non-high-density lipoprotein cholesterol (non-HDL-C), total cholesterol, HDL-C, apolipoprotein B, lipoprotein (a) [Lp(a)], and triglycerides; the proportion of patients achieving LDL-C < 70 mg/dl (1.81 mmol/L) or < 100 mg/dl (2.59 mmol/L); and the proportion of patients with LDL-C < 70 mg/dl and/or a ≥ 50 % reduction in calculated LDL-C.

On-site assessments were performed at baseline (week 0) and then weeks 12, 24, 36, 52, 64, 78, and the end-of-study visit, week 86, for patients not entering the open-label extension study. Fasting blood samples for lipid analysis were collected in the morning. Lipid analyzes were performed at a central laboratory and LDL-C was calculated using the Friedewald formula [28].

### Safety

Safety was assessed by monitoring adverse events (AEs), serious AEs, laboratory parameters, vital signs, and anti-alirocumab antibody development. Treatment emergent AEs (TEAEs) were defined as AEs that occurred, worsened, or became serious during the period from first to last injection (+70 days), or to the first injection in the open-label extension study, whichever came first.

AEs of special interest included allergic events, local injection-site reactions, hemolytic anemia, increases in alanine aminotransferase, neurologic disorders, neurocognitive events, ophthalmological disorders, and adjudicated cardiovascular events (categorized as coronary heart disease death, non-fatal myocardial infarction, fatal and non-fatal ischemic stroke, unstable angina requiring hospitalization and congestive heart failure requiring hospitalization). Pre-dose samples were obtained at baseline and several time points up to the end of study for assessment of anti-alirocumab antibodies, performed by the Regeneron Clinical Bioanalysis Group (Regeneron Pharmaceuticals, Inc. Tarrytown, NY, USA).

### Statistical Analysis

A sample size of 45 patients was calculated to have 95 % power to detect a difference of at least 30 % in mean percent change from baseline in LDL-C at week 24 with 5 % two-sided significance level, assuming a common standard deviation of 25 % (based on previous experience with alirocumab [16]). Nevertheless, to meet regulatory requirements across the program, this sample size was increased to 105 patients to assess the safety of alirocumab.

The primary efficacy endpoint was analyzed in the ITT population, defined as all randomized patients with at least one calculated LDL-C value at baseline and at least one available calculated LDL-C value within one of the analysis windows up to week 24. A pre-specified on-treatment analysis was also performed, which excluded patients from the ITT population lacking an LDL-C value obtained while receiving study treatment (up to 21 days after last injection). A mixed effect model with repeated measures approach to account for missing data [29, 30] was used to provide least-squares (LS) means estimates within each treatment arm and for the comparison between treatment arms for the primary and continuous secondary efficacy endpoints, (except Lp(a) and triglycerides), with a hierarchical procedure used to control type I error and handle multiple secondary endpoint analyzes. Lp(a) and triglycerides were analyzed using a multiple imputation approach to handle missing data, followed by robust regression [31]. The proportion of patients achieving pre-defined LDL-C goals was analyzed using a multiple imputation approach to handle missing data followed by stratified conditional logistic regression model with treatment group as the main effect, baseline LDL-C value as covariate, and stratified by the randomization factors. A sensitivity analysis of the primary endpoint using a pattern mixture model was also conducted to assess the robustness of used statistical methods. Missing calculated LDL-C values during the on-treatment period were multiply imputed using a model assuming ‘missing at random’, and missing calculated LDL-C values during the post-treatment period were multiply imputed using random draws from a normal distribution where the mean was equal to the subject’s own baseline value.

An additional post-hoc sensitivity analysis was conducted excluding patients from three study sites with serious Good Clinical Practice non-compliance.

All randomized and treated patients were included in the safety analyzes. Safety data were analyzed by descriptive statistics. All statistical analyzes were conducted using SAS® (SAS® Institute Inc., Cary, NC).

All lipid measurements and clinical laboratory tests were performed using standard procedures by a central laboratory (Medpace), which was standardized by, and participated in, the CDC-NHLBI part III Lipid Standardization Program [32].

## Results

### Patients

In total, 107 eligible patients were randomized to treatment with alirocumab (*n* = 72) or placebo (*n* = 35) (Fig. 1). Baseline characteristics and lipid parameters of randomized patients (Table 1) were generally similar between the groups, although some imbalances were observed in terms of gender, proportion of patients with coronary heart disease or risk equivalents, and patients receiving ezetimibe as additional background LLT. In both groups, all patients received background statin therapy, with 72.9 % of all patients receiving high-intensity statin. In addition, 27.1 % of patients were also receiving other LLTs (overall, 24.3 % of patients were receiving ezetimibe [19.4 % alirocumab; 34.3 % placebo]). A total of 73.6 % of patients in the alirocumab group and 77.1 % in the placebo group completed the double-blind treatment period (at least 76 weeks of exposure and visit week 78 performed). Over the 78-week treatment period, the compliance to study medication was similar between treatment groups (97.8 % alirocumab; 96.9 % placebo). Three of 33 study sites, with a total of 20 randomized patients (alirocumab: *n* = 15; placebo: *n* = 5), were closed due to serious breach of compliance with Good Clinical Practice.

### Efficacy

**Fig. 2:**
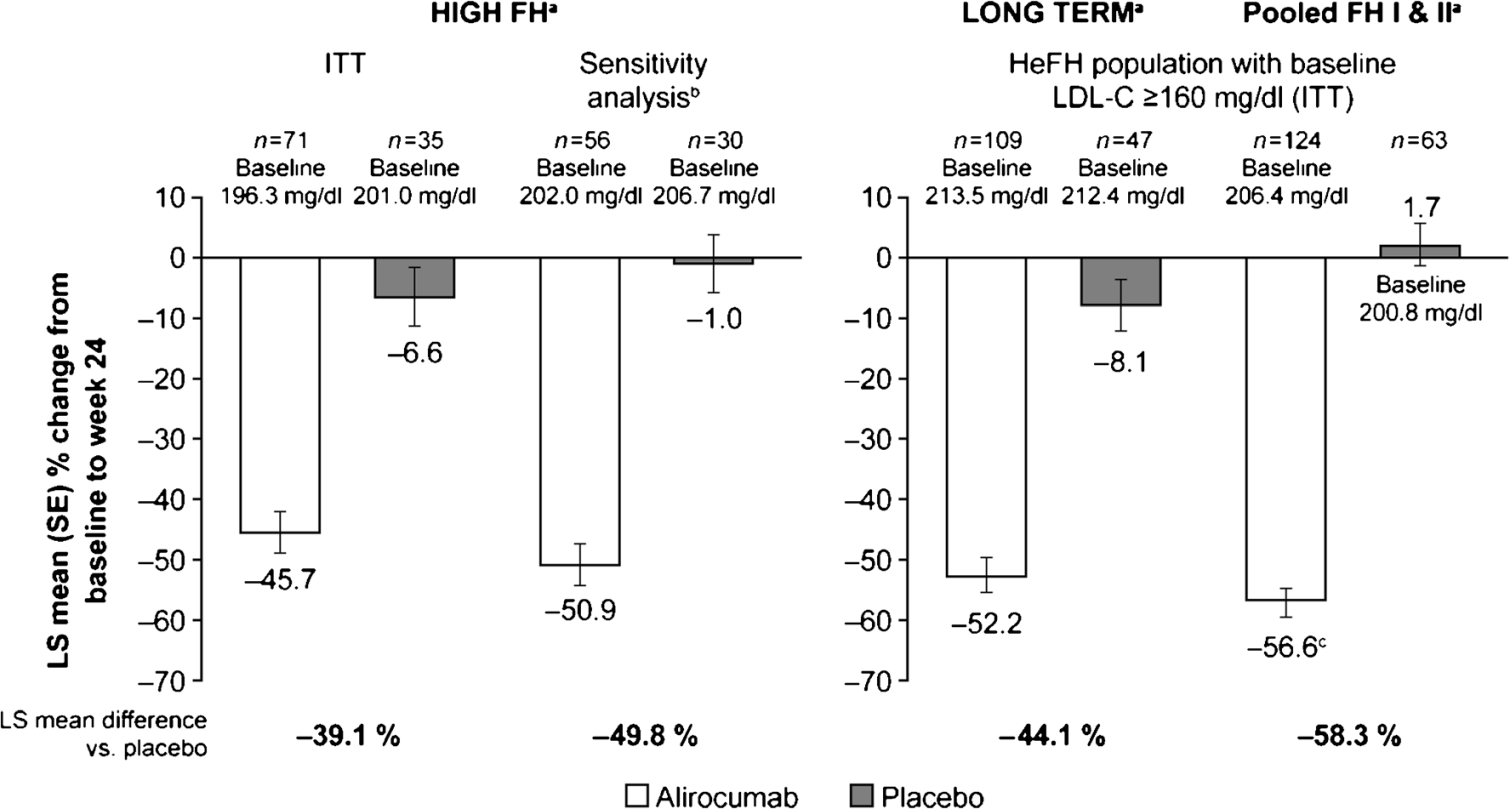
LDL-C percent change from baseline to week 24: comparison with patients with heFH from ODYSSEY LONG TERM and patients from ODYSSEY FH I and II with LDL-C baseline ≥ 160 mg/dl ^a^All patients were on background of maximally tolerated statin ± other LLT ^b^Excluding non-Good Clinical Practice compliant sites ^c^A total of 70.9 % (FH I) and 80.0 % (FH II) of the alirocumab-treated patients with baseline LDL-C ≥ 160 mg/dl received dose adjustment from 75 mg Q2W to 150 mg Q2W at week 12. ITT, intent-to-treat; LLT, lipid-lowering therapy; LS, least squares; SE, standard error

At week 24, the LS mean (standard error [SE]) percent change in LDL-C from baseline was greater in the alirocumab add-on group (−45.7 [3.5] %) compared with the placebo group (−6.6 [4.9] %) in the ITT population, with a statistically significant LS mean (SE) difference between the groups of −39.1 (6.0) % (*P* < 0.0001; Table 2 and Fig. 2). The results for the on-treatment analysis were similar at week 24 (alirocumab −45.5 [3.5] %; placebo −6.6 [5.0] %; *P* < 0.0001) (Supplementary Table 1). A sensitivity analysis was conducted excluding patients from the three non-compliant sites and demonstrated LS mean (SE) percent decrease from baseline to week 24 with alirocumab (−50.9 [3.4] %) vs. placebo (−1.0 [4.7] %) in LDL-C (LS mean difference vs. placebo −49.8 [5.8] %; *P* < 0.0001) (Fig. 2 and Supplementary Table 2).

**Table 2:** Effect of alirocumab versus placebo on LDL-C, secondary lipid parameters, and achievement of LDL-C target levels at week 24 (ITT analysis)

All patients on background of maximally tolerated statin ± other lipid-lowering therapy	Alirocumab 150 mg Q2W (*n* = 71)	Placebo (*n* = 35)	Alirocumab vs. placebo		
			Difference vs. placebo	95 % CI	*P* value
Week 24					
Absolute LDL-C level (SE), mg/dl	107.0 (6.7)	182.3 (9.5)			
Absolute change (SE) from baseline, mg/dl	−90.8 (6.7)	−15.5 (9.5)	−75.3 (11.6)	−98.4 to −52.2	<0.0001
LS mean (SE) change in calculated LDL-C from baseline to week 24	−45.7 (3.5)	−6.6 (4.9)	−39.1 (6.0)	−51.1 to −27.1	<0.0001*
Proportion of very high CV risk patients reaching calculated LDL-C < 70 mg/dl or high CV risk patients reaching calculated LDL-C < 100 mg/dl, %	41.0^a^	5.7^a^			0.0016*
% change from baseline to week 24 in other lipid parameters, LS mean (SE)					
Non-HDL-C	−41.9 (3.1)	−6.2 (4.3)	−35.8 (5.3)	−46.3 to −25.3	<0.0001*
ApoB	−39.0 (2.7)^b^	−8.7 (3.8)^c^	−30.3 (4.7)	−39.7 to −20.9	<0.0001*
Total cholesterol	−33.2 (2.6)	−4.8 (3.6)	−28.4 (4.4)	−37.3 to −19.6	<0.0001*
Lp(a)^d^	−23.5 (3.7)	−8.7 (5.0)	−14.8 (6.2)	−26.9 to −2.7	0.0164*
HDL-C	7.5 (1.9)	3.9 (2.7)	3.7 (3.3)	−2.9 to 10.2	0.2745
TGs^d^	−10.5 (3.3)	−1.9 (4.8)	−8.7 (5.9)	−20.2 to 2.8	0.1386

The *P* value is followed by a ‘*’ if statistically significant according to the fixed hierarchical approach used to ensure a strong control of the overall type-I error rate at the 0.05 level

*Apo*, apolipoprotein; *CI*, confidence interval; *CV*, cardiovascular; *CVD*, cardiovascular disease; *HDL-C*, high-density lipoprotein cholesterol; *ITT*, intent-to-treat; *LDL-C*, low-density lipoprotein cholesterol; *Lp(a)*, lipoprotein(a); *LS*, least squares; *Q2W*, every 2 weeks; *SE*, standard error; *TGs*, triglycerides

^a^Combined estimate for proportion of patients reaching the level

^b^ *n* = 69

^c^ *n* = 34

^d^Combined estimate for adjusted proportion

**Fig. 3:**
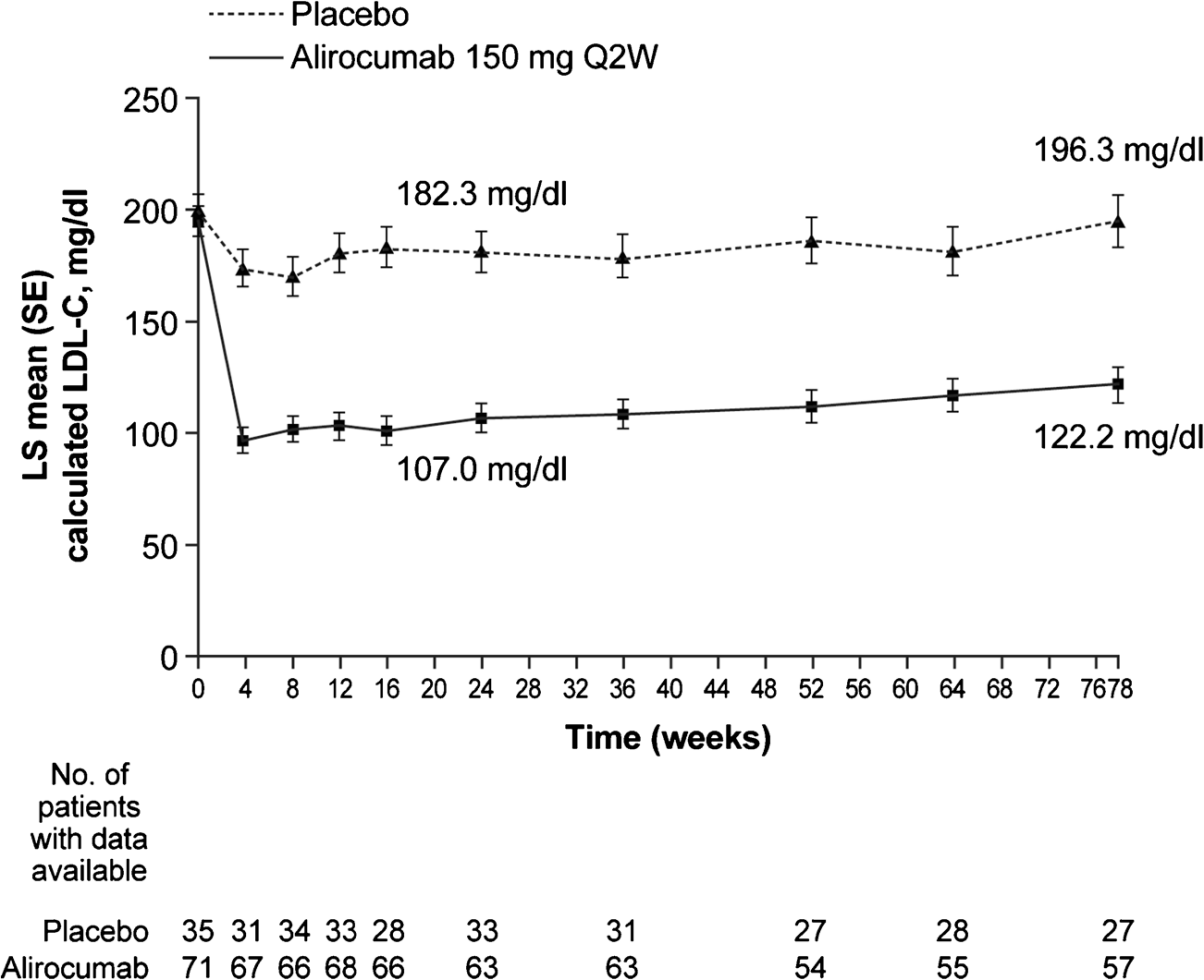
LDL-C levels over time (ITT analysis). Values above data points indicate calculated LDL-C (LS mean, mg/dl). All patients on background of maximally tolerated statin ± other lipid-lowering therapy. LDL-C, low-density lipoprotein cholesterol; LS, least squares; Q2W, every 2 weeks; SE, standard error

The reduction in LDL-C with alirocumab allowed 41.0 % of patients to achieve their pre-defined LDL-C target levels of < 70 or 100 mg/dl (depending on cardiovascular risk) at week 24, compared with only 5.7 % of patients in the placebo arm; LDL-C levels of < 70 mg/dl were achieved by 32.4 % and 2.9 % of patients, respectively (ITT analysis). In the on-treatment analysis, 50.7 % of patients in the alirocumab arm versus 11.4 % in the placebo arm reached a calculated LDL-C level of < 100 mg/dl, regardless of risk at week 78. The calculated LDL-C levels over time in the alirocumab and placebo arms from baseline to week 78 are shown in Fig. 3: alirocumab maintained consistent LDL-C reductions over the course of the study, with the reductions observed by week 4 remaining largely constant through 78 weeks of treatment. The LS mean (SE) absolute reduction in LDL-C levels from baseline to week 24 with alirocumab was 90.8 (6.7) mg/dl (75.3 [11.6] mg/dl vs. placebo).

A sensitivity analysis of the primary endpoint using the pattern mixture model to handle missing data showed consistent results with the primary analysis (Supplementary Table 3).

**Table 3:** AEs and safety laboratory values (safety population)

All patients on background of maximally tolerated statin ± other lipid-lowering therapy	Alirocumab 150 mg Q2W (*n* = 72)	Placebo (*n* = 35)
TEAEs,^a^ % (n)	70.8 (51)	80.0 (28)
Treatment-emergent SAEs, % (n)	13.9 (10)	11.4 (4)
TEAEs leading to death, % (n)	0	0
TEAEs leading to treatment discontinuation, % (n)	4.2 (3)	5.7 (2)
AEs of interest		
Any local injection-site reaction,^b^ % (n)	8.3 (6)	5.7 (2)
Mild intensity	83.3 (5)	100 (2)
Moderate intensity	16.7 (1)	0
Severe intensity	0	0
Potential general allergic events, % (n)	6.9 (5)	5.7 (2)
Neurological events, % (n)	2.8 (2)	2.9 (1)
Neurocognitive disorders, % (n)	1.4 (1)	2.9 (1)
Ophthalmologic disorders, % (n)	1.4 (1)	0
Positively adjudicated CV events, % (n)	8.3 (6)	0
CHD death	0	0
Non-fatal MI	5.6 (4)	0
Ischemic stroke	0	0
Unstable angina requiring hospitalization	0	0
CHF requiring hospitalization	1.4 (1)	0
Ischemia-driven coronary revascularization procedure	6.9 (5)	0
Type II diabetes	1.4 (1)	2.9 (1)
Hepatic disorders	5.6 (4)	8.6 (3)
Laboratory values		
Alanine aminotransferase >3 times ULN	4.2 (3)	2.9 (1)
Aspartate aminotransferase >3 times ULN	1.4 (1)	0
Creatine kinase >3 times ULN	2.8 (2)^c^	2.9 (1)

*AE*, adverse event; *CHD*, coronary heart disease; *CHF*, coronary heart failure; *CV*, cardiovascular; *MI*, myocardial infarction; *Q2W*, every 2 weeks; *SAE*, serious adverse event; *TEAE*, treatment-emergent adverse event; *ULN*, upper limit of normal

^a^TEAEs are AEs that developed or worsened or became serious during the TEAE period (time from first dose of double-blind treatment to last dose +70 days or first dose of open-label treatment, whichever came first). More than one TEAE may be reported per patient

^b^In case of several occurrences, the maximal intensity was used

^c^ *n* = 71

At week 24, significant reductions from baseline versus placebo were also observed in non-HDL-C (alirocumab: −41.9 [3.1] %; placebo: −6.2 [4.3] %; *P* < 0.0001), total cholesterol (alirocumab: −33.2 [2.6] %; placebo: −4.8 [3.6] %; *P* < 0.0001), apolipoprotein B (alirocumab: −39.0 [2.7] %; placebo: −8.7 [3.8] %; *P* < 0.0001), and Lp(a) (alirocumab: −23.5 [3.7] %; placebo: −8.7 [5.0] %; *P* = 0.0164) in patients receiving alirocumab (Table 2). No significant changes were observed for either HDL-C (alirocumab: 7.5 [1.9] %; placebo: 3.9 [2.7] %) or triglyceride levels (alirocumab: −10.5 [3.3] %; placebo: −1.9 [4.8] %) (Table 2). At week 78, reductions with alirocumab compared with placebo were observed for Lp(a), non-HDL-C, apolipoprotein B, and total cholesterol (Supplementary Table 4).

### Safety

Overall, 70.8 % of patients in the alirocumab group experienced a TEAE compared with 80.0 % in the placebo group (Table 3). There were no reported deaths. A total of five patients prematurely discontinued the treatment following at least one TEAE (three patients [4.2 %] in the alirocumab add-on group and two patients [5.7 %] in the placebo group) with no specific clinical pattern. The percentages of patients experiencing treatment-emergent serious AEs were similar in the alirocumab (13.9 %) and placebo (11.4 %) groups (Table 3). The TEAEs occurring in ≥2 % of patients in either the alirocumab or placebo group are shown in Supplementary Table 5.

Considering the TEAEs of special interest, a similar proportion of patients in both groups experienced potential general allergic (alirocumab 6.9 % [*n* = 5]; placebo 5.7 % [*n* = 2]) and neurological events (alirocumab 2.8 % [*n =* 2]; placebo 2.9 % [*n* = 1]) (Table 3). One patient in each group reported a neurocognitive event: disturbance in attention in one alirocumab-treated patient (1.4 %) and amnesia in one placebo patient (2.9 %). Injection-site reactions were reported by 8.3 % (*n* = 6) of patients in the alirocumab group (vs. 5.7 % [*n* = 2] placebo); most were mild in severity and did not result in study medication discontinuation. One patient in the alirocumab-treated group experienced an ophthalmological TEAE (chorioretinopathy). The investigator and sponsor considered the event not to be related to the investigational medicinal product, statin, or other LLT. No cases of confirmed hemolytic anemia were reported. Hepatic disorders were experienced by a similar proportion of patients in the alirocumab and placebo groups (5.6–8.6 % [*n* = 3–4]). TEAEs related to the worsening or development of diabetes (diabetes mellitus or diabetic complication) were reported in one patient in each treatment group (alirocumab: 1.4 %; placebo: 2.9 %). Adjudicated treatment-emergent cardiovascular events were reported in six (8.3 %) alirocumab-treated patients (vs. no placebo patients) as follows: non-fatal myocardial infarction (*n* = 4), coronary heart failure requiring hospitalization (*n* = 1) and ischemia-driven coronary revascularization procedure (*n* = 5).

A total of four patients (5.6 %) in the alirocumab-treated group had LDL-C values of < 25 mg/dl (0.65 mmol/L) on at least two consecutive occasions; one of those patients experienced two consecutive LDL-C values < 15 mg/dl (0.39 mmol/L). One patient experienced chorioretinopathy at week 44, 10 months after the first study drug administration, during which the LDL-C level remained < 25 mg/dl from weeks 4–24 and was at 53 mg/dl at week 52. No other specific safety concerns were identified in patients with LDL-C values of < 15 or < 25 mg/dl.

### Anti-Alirocumab Antibodies

Administration of alirocumab 150 mg Q2W for 78 weeks was associated with low levels of immunogenicity. No patients had pre-existing immunoreactivity. Positive responses in the anti-drug antibody (ADA) assay were observed in two patients, one in each group (alirocumab: 1/50 [2.0 %], placebo 1/29 [3.4 %]). In the alirocumab patient, the ADA assay response was transient, detected at a single time point (week 52), and drug efficacy was not affected since the LDL-C reduction from baseline was maintained at 40 % over the study duration including at week 52. Furthermore, the ADA assay response in this patient was very low (minimum titer in the assay). In the patient from the placebo group, a positive ADA assay response was observed at weeks 52 and 78. Since this patient was not administered alirocumab, this signal was most likely due to high serum background levels in the ADA assay and not a drug-induced ADA response. None of the samples positive in the ADA assay were neutralizing.

## Discussion

In this study of patients with heFH and very high baseline levels of LDL-C, despite maximally tolerated statins ± other LLT, alirocumab 150 mg Q2W demonstrated significant reductions in LDL-C levels compared with placebo, achieving a mean absolute LDL-C reduction of 90.8 mg/dl at week 24.

The LDL-C reduction from baseline to week 24 in the current study was −45.7 % with alirocumab 150 mg Q2W (vs. placebo: −6.6 %); this compared with a reduction of −52.2 % with alirocumab 150 mg Q2W (placebo: −8.1 %) in the subset of patients with heFH from ODYSSEY LONG TERM with high baseline LDL-C levels of ≥ 160 mg/dl (the changes from baseline for the overall study population in LONG TERM were −61.0 % for alirocumab and 0.8 % for placebo) (Fig. 2). In a separate pool of patients with baseline LDL-C levels of ≥ 160 mg/dl from studies ODYSSEY FH I & II, alirocumab 75 mg Q2W (with possible dose adjustment to 150 mg Q2W) demonstrated a slightly higher LDL-C reduction (alirocumab: −56.6 %; placebo: 1.7 %) (Fig. 2). Significant reductions in LDL-C levels have also been seen with a bi-weekly or four-weekly dosing regimen of another PCSK9 inhibitor, evolocumab, in patients with HeFH and either with or without statin intolerance [12, 13, 33–35].

The HIGH FH study was designed to assess the efficacy and safety in a specific group of patients with a particularly high level of LDL-C at screening and a sufficient number of patients were included in the current study for the purposes of assessing the primary endpoint, LDL-C percentage change at week 24 for alirocumab versus placebo (−39.1 %, *P* < 0.0001). This was lower than reductions seen in other studies in the overall ODYSSEY program, including those with FH patients, and it is important to consider the potential reasons for this difference. One possibility is that patients enrolled in HIGH FH differed from HeFH patients in other ODYSSEY trials [19]. For example, a smaller proportion of HIGH FH patients were on high-intensity statin and ezetimibe compared with HeFH patients in other ODYSSEY studies [19]. We are unsure as to why this occurred, although intolerance to ezetimibe could have been one reason for these patients having LDL-C levels of 160 mg/dL or greater. Whatever the reason, generalization of these findings with respect to efficacy should be viewed with this difference in background therapy taken into consideration. Additionally, the relatively low number of patients randomized to placebo (*n* = 35) resulted in a higher variability in results which might not be seen in a larger study. In fact, the placebo-corrected observations of a number of participants were influenced by significant reductions in the placebo arm. Finally, three of the study sites (with 20 randomized patients) were closed due to serious breaches of compliance with Good Clinical Practice; when these sites were omitted in a sensitivity analysis, greater reductions in LDL-C levels from baseline were seen with alirocumab (−49.8 % vs. placebo; alirocumab: −50.9 %, placebo: −1.0 %; a difference of over 10 percentage points compared with the primary analysis).

The small size of HIGH FH also does not allow for conclusive safety observations to be made, in particularly for the rare adverse events. As a consequence, safety results should be interpreted with caution and must be considered in the context of larger studies and the ODYSSEY program as a whole.

With LDL-C baseline levels of 196.3–201.0 mg/dl in patients on maximum-tolerated statin ± other LLT, this study population reflected one at significantly increased risk of cardiovascular events and in need of further treatment to reduce LDL-C. Importantly, these patients with heFH receiving alirocumab achieved a mean absolute LDL-C reduction of 90.8 mg/dl at week 24 in patients with heFH. These large reductions in LDL-C levels were observed from week 4 to end of treatment, suggesting a sustained LDL-C-lowering effect with alirocumab. Despite very high baseline LDL-C levels, 41.0 % of patients receiving alirocumab achieved their pre-defined LDL-C goals of < 70 mg/dl or < 100 mg/dl, depending on cardiovascular risk. Regardless of prior cardiovascular events, 32.4 and 57.0 % of patients reached the LDL-C levels of < 70 and < 100 mg/dl, respectively, which have been defined as treatment targets in FH guidelines [1, 2, 36–38].

Incremental lowering of LDL-C levels to below previously defined targets with the addition of ezetimibe to statin therapy has been demonstrated to improve cardiovascular outcomes [5]. Several large clinical outcomes studies with PCSK9 monoclonal antibodies including alirocumab [39] are ongoing. Compared with ezetimibe, alirocumab provides a greater incremental reduction in LDL-C levels when added to statin therapy [20, 21]; the results of the ongoing clinical ODYSSEY OUTCOMES study (NCT01663402) will help to establish whether this also translates into a beneficial reduction in cardiovascular events [39]. Furthermore, consistent with previous ODYSSEY phase 3 studies [19–21, 24, 25], alirocumab significantly reduced Lp(a) levels; an additional benefit not observed with ezetimibe treatment when added to statin therapy [5].

The incidence of TEAEs was broadly similar in the alirocumab-treated and placebo groups, with a comparable incidence of AEs leading to treatment discontinuation between both groups. The overall rate of AEs of special interest was low, including TEAEs related to allergic events, as well as hepatitis, ophthalmologic, neurologic, and neurocognitive disorders. There was an imbalance in treatment-emergent cardiovascular events confirmed by adjudication, which were reported by six alirocumab-treated patients (8.3 %) compared with no placebo patients. It should be noted, however, that the current study is not adequately powered to look at differences in adjudicated cardiovascular events and the number of patients randomized in HIGH FH was small (*n* = 107). In a previous study with 2341 randomized patients treated for 78 weeks, alirocumab indicated a reduction of cardiovascular events in a post-hoc analysis [25]; however, additional data are required to confirm this observation. Safety across all alirocumab phase 2 and 3 trials has been analyzed in a large pooled database, and cardiovascular outcomes are being formally assessed in the ongoing 18,000-patient ODYSSEY OUTCOMES study [39]. No deaths occurred during the study. No cases of chorioretinopathy have been previously reported in the other trials in the ODYSSEY program [20, 21, 23–25]. The safety data are consistent with previous phase 3 ODYSSEY studies demonstrating no excess of AEs in alirocumab-treated patients [20, 21, 23–25].

In conclusion, this study showed that self-administered alirocumab 150 mg Q2W in patients with heFH and LDL-C ≥ 160 mg/dl at baseline produced a significant decrease in LDL-C from baseline to week 24 versus placebo (LDL-C decrease of 90.8 mg/dl) on top of statin and other LLT with a compliance of 98 % to study medication at week 78. Alirocumab was generally well tolerated, with TEAEs comparable with placebo. These findings further support the use of alirocumab as a therapeutic option in patients with heFH, potentially allowing for reduction in LDL-C to levels that were previously unobtainable using existing standard of care therapies. Ongoing clinical outcomes studies of PCSK9 monoclonal antibodies will help to establish potential clinical benefits of these therapies.

## Electronic supplementary material

ESM 1 (DOCX 89 kb)
