# Supplementary material for: Efficacy and Safety of Alirocumab in Patients with Heterozygous Familial Hypercholesterolemia and LDL-C of 160 mg/dl or Higher
Source: Cardiovasc Drugs Ther. 2016 Sep 13;30(5):473–83. doi: 10.1007/s10557-016-6685-y (PMC5055560; doi:10.1007/s10557-016-6685-y)
Supplement: Supplementary file 1 — (DOCX 89 kb) [file 10557_2016_6685_MOESM1_ESM.docx]

# Efficacy and Safety of Alirocumab in Patients with Heterozygous Familial Hypercholesterolemia and LDL-C of 160 mg/dl or Higher

Henry N. Ginsberg • Daniel J. Rader • Frederick J. Raal • John R. Guyton • Marie T. Baccara-Dinet • Christelle Lorenzato • Robert Pordy • Erik Stroes

Submitted to Cardiovascular Drugs and Therapy

- Corresponding author:
  Professor Henry N. Ginsberg, Columbia University College of Physicians and Surgeons, Irving Institute for Clinical and Translational Research, Columbia University, 622 West 168th Street, New York, NY 10032
  Tel: +1 212 305 9562; fax: +1 212 305 3213; e-mail address: [hng1@cumc.columbia.edu](mailto:hng1@cumc.columbia.edu)

Contents

[Supplementary materials: HIGH FH Investigators 3](#_Toc435183377)

[Supplementary Materials: Methods 5](#_Toc435183378)

[Selection of preferred terms for the adverse events of special interest categories (based on Standardized Medical Dictionary of Regulatory Activities [MedDRA] Queries) 5](#_Toc435183379)

[Supplementary Table 1 Effect of alirocumab versus placebo on LDL-C at week 24 (on-treatment analysis) 6](#_Toc435183380)

[Supplementary Table 2 Sensitivity analysis of primary endpoint (% change from baseline to week 24 in LDL-C) excluding three sites with serious Good Clinical Practice non-compliance 7](#_Toc435183381)

[Supplementary Table 3 Percent change from baseline in calculated LDL-C at week 24: pattern-mixture model (randomized population) 8](#_Toc435183382)

[Supplementary Table 4 (a–g) Effect of alirocumab versus placebo on (a) LDL-C, (b) non-HDL-C, (c) ApoB, (d) total cholesterol, (e) Lp(a), (f) HDL-C and (g) TGs at weeks 12, 24, 52, and 78 (ITT analysis) 9](#_Toc435183383)

[Supplementary Table 5 TEAEs^a^ occurring in ≥2 % patients (safety population) 16](#_Toc435183384)

[Supplementary Fig 1 ODYSSEY HIGH FH study design 23](#_Toc435183385)

# Supplementary materials: HIGH FH Investigators

**Canada:** P. Perron (Sherbrooke, Quebec); J. Bergeron (Sainte-Foy, Quebec).

**United States:** H. Ginsberg (New York, NY); J. Guyton (Durham, NC); J. Homan (Newport Beach, CA); M. Cuchel (Philadelphia, PA); R. Schramm (Ponte Vedra, FL); R. Greenfield (Fountain Valley, CA); T. Turner (Cincinnati, OH); L. Martin (Washington, DC); F. Dunn (Dallas, TX); R. DeLuca (Miami, FL); G. Salem (Bell Gardens, CA).

**Netherlands:** E.S.G. Stroes (Amsterdam); S.H.J. Donders (Groningen); M.A. van de Ree (Utrecht); J.P.R. Herrman (Amsterdam); M.V. Huisman (Leiden).

**Russia:** E. Demchenko (St. Petersburg); A. Lebedeva (Moscow); G. Kamalov (Kazan); V. Arkhipovsky (Arkhangelsk); V. Marasaev (Yaroslavl); V. Gurevich (St. Petersburg); A. Susekov (Moscow).

**South Africa:** F.C.J. Bester (Bloemfontein); F. Raal (Johannesburg); S. Middlemost (Western Cape); N. Fourie (Bloemfontein); M. Pretorius (Cape Town).

**Steering Committee:** *Chairman:* H. Ginsberg (Irving Institute for Clinical and Translational Research, Columbia University New York, NY, USA). *Members:* J.G. Robinson (The University of Iowa, Iowa City, IA, USA); D.J. Rader (Institute for Translational Medicine and Therapeutics, University of Pennsylvania School of Medicine Philadelphia, PA, USA); C.P. Cannon (TIMI Study Group; Harvard Clinical Research Institute, Boston, MA USA); H. Colhoun (Clinical Centre, University of Dundee, Ninewells Hospital & Medical School, Dundee, UK); J.J.P. Kastelein (Academic Medical Center University of Amsterdam, Amsterdam, The Netherlands); M. Farnier (Point Médical, Département d’Endocrinologie et de Lipidologie, Dijon, France); Y. Huo (Peking University First Hospital, Beijing, China).

**Data Monitoring Committee** *DMC Chairman:* A. Olsson (Bromma, Sweden). *Members:* D. Waters (Division of Cardiology, San Francisco General Hospital, San Francisco, CA, USA); D. Larrey (Hôpital Saint-Eloi Service d’hépato-gastroentérologie, Montpellier, France); R.S. Rosenson (Director, Cardiometabolic Disorders, Mount Sinai Heart; Professor of Medicine, Mount Sinai School of Medicine, New York, NY, USA); P.A. Patriarca (Biologics Consulting Group, Inc., Alexandria, VA, USA); G. Molenberghs (Center for Statistics [CenStat] Universiteit Hasselt, Diepenbeek, Belgium).

**Clinical Events Committee (Reviewers)**, **Duke Clinical Research Institute, Durham, NC USA**; P. Tricoci, CEC Principal Investigator, Cardiology; K.W. Mahaffey, CEC Director, Cardiology; R.D. Lopes, Cardiology; B.R. Shah, Cardiology; R.H. Mehta, Cardiology; M.T. Roe, Cardiology; Z. Eapen, Cardiology; L. Armaganijan, Cardiology; A. Bertolami, Cardiology; S. Leonardi, Cardiology; B.J. Kolls, Neurology; J.D. Jordan, Neurology; Grégory Ducrocq, Cardiology; E. Puymirat, Cardiology; R. Mathews, Cardiology.

**Independent physicians monitoring two consecutive LDL <0.65 mmol/L:** Karen Alexander and Chiara Melloni (Duke Clinical Research Institute, Durham, NC USA).

# Supplementary Materials: Methods

## Selection of preferred terms for the adverse events of special interest categories (based on Standardized Medical Dictionary of Regulatory Activities [MedDRA] Queries)

Potential general allergic events: 'hypersensitivity' (broad + narrow) excluding the following preferred terms: 'infusion-site dermatitis', 'infusion-site hypersensitivity', 'infusion-site rash', 'infusion site urticaria', 'injection-site dermatitis', 'injection-site hypersensitivity', 'injection-site rash', 'injection-site urticaria', and 'injection-site vasculitis'.

Neurological events: 'demyelination' (broad + narrow), 'peripheral neuropathy' (broad + narrow), and 'Guillain-Barre syndrome' (broad + narrow) excluding 'acute respiratory distress syndrome', 'asthenia', 'respiratory arrest', and 'respiratory failure'.

Neurocognitive disorders: selected with the use of a custom MedDRA query that was based on the following five High-Level Group terms: “deliria (including confusion)”, “cognitive and attention disorders and disturbances”, “dementia and amnestic conditions”, “disturbances in thinking and perception”, and “mental impairment disorders”.

Ophthalmologic disorders: 'optic nerve disorders' (broad + narrow), 'retinal disorders' (narrow), and 'corneal disorders' (narrow)

Diabetes: using the custom MedDRA query “diabetes”, which includes: “diabetes complications”, High-Level Group Terms “diabetes mellitus” and “carbohydrate tolerance analyses (including diabetes)”, High-Level Terms (excluding preferred terms “blood glucose decreased”), and “hyperglycaemia”.

# Supplementary Table 1 Effect of alirocumab versus placebo on LDL-C at week 24 (on-treatment analysis)

| All patients on background of maximally tolerated statin ± other lipid-lowering therapy | Alirocumab 150 mg Q2W (*n*=71) | Placebo (*n*=35) |
| --- | --- | --- |
| Baseline LDL-C, mean (SD), mg/dl | 196.3 (57.9) | 201.0 (43.4) |
| Week 24 % change from baseline, LS mean (SE) | –45.5 (3.5) | –6.6 (5.0) |
| LS mean difference (SE) vs. placebo | –38.9 (6.1) |  |
| 95% CI | (–51.0 to –26.9) |  |
| *P* value vs. placebo | <0.0001* |  |

The *P* value is followed by a '*' if statistically significant according to the fixed hierarchical approach used to ensure a strong control of the overall type-I error rate at the 0.05 level.

CI, confidence interval; LDL-C, low-density lipoprotein cholesterol; LS, least squares; Q2W, every 2 weeks; SD, standard deviation; SE, standard error

# Supplementary Table 2 Sensitivity analysis of primary endpoint (% change from baseline to week 24 in LDL-C) excluding three sites with serious Good Clinical Practice non-compliance

| All patients on background of maximally tolerated statin ± other lipid-lowering therapy | Alirocumab 150 mg Q2W (*n*=56) | Placebo (*n*=30) |
| --- | --- | --- |
| Baseline LDL-C, mean (SD), mg/dl | 202.0 (56.7) | 206.7 (44.0) |
| Week 24 % change from baseline, LS mean (SE) | –50.9 (3.4) | –1.0 (4.7) |
| LS mean difference (SE) vs. placebo | –49.8 (5.8) |  |
| 95% CI | (–61.3 to –38.3) |  |
| *P* value vs. placebo | <0.0001 |  |

The P value is not adjusted for multiplicity and provided for descriptive purpose only

CI, confidence interval; LDL-C, low-density lipoprotein cholesterol; LS, least squares; Q2W, every 2 weeks; SD, standard deviation; SE, standard error

# Supplementary Table 3 Percent change from baseline in calculated LDL-C at week 24: pattern-mixture model (randomized population)

| All patients on background of maximally tolerated statin ± other lipid-lowering therapy | Alirocumab 150 mg Q2W (*n*=72) | Placebo (*n*=35) |
| --- | --- | --- |
| Baseline LDL-C, mean (SD), mg/dl | 196.3 (57.9) | 201.0 (43.4) |
| Week 24 % change from baseline, LS mean (SE) | –45.7 (3.5) | –6.8 (5.0) |
| LS mean difference (SE) vs. placebo | –38.8 (6.1) |  |
| 95% CI | (–51.0 to –26.7) |  |
| *P* value vs. placebo | <0.0001 |  |

The *P* value is not adjusted for multiplicity and provided for descriptive purpose only

CI, confidence interval; LDL-C, low-density lipoprotein cholesterol; LS, least squares; Q2W, every 2 weeks; SD, standard deviation; SE, standard error

# Supplementary Table 4 (a–g) Effect of alirocumab versus placebo on (a) LDL-C, (b) non-HDL-C, (c) ApoB, (d) total cholesterol, (e) Lp(a), (f) HDL-C and (g) TGs at weeks 12, 24, 52, and 78 (ITT analysis)

1. **LDL-C**

| All patients on background of maximally tolerated statin ± other lipid-lowering therapy | Alirocumab  150 mg Q2W (*n*=71) | Placebo (*n*=35) | Alirocumab vs. placebo | | |
| --- | --- | --- | --- | --- | --- |
|  |  |  | Difference vs. placebo | 95% CI | *P* value |
| **LS mean (SE) % change in calculated LDL-C from baseline** | | | | | |
| Week 12 | –46.9 (3.2) | –6.6 (4.6) | –40.3 (5.6) | –51.4 to  –29.3 | <0.0001* |
| Week 24 | –45.7 (3.5) | –6.6 (4.9) | –39.1 (6.0) | –51.1 to –27.1 | <0.0001* |
| Week 52 | –42.1 (4.2) | –3.0 (5.9) | –39.1 (7.3) | –53.6 to  –24.6 | <0.0001* |
| Week 78 | –37.9 (4.5) | 1.2 (6.4) | –39.0 (7.8) | –54.6 to  –23.5 | <0.0001 |

The *P* value is followed by a '*' if statistically significant according to the fixed hierarchical approach used to ensure a strong control of the overall type-I error rate at the 0.05 level

CI, confidence interval; LDL-C, low-density lipoprotein cholesterol; LS, least squares; Q2W, every 2 weeks; SD, standard deviation; SE, standard error

1. **Non-HDL-C**

| All patients on background of maximally tolerated statin ± other lipid-lowering therapy | Alirocumab  150 mg Q2W (*n*=71) | Placebo (*n*=35) | Alirocumab vs. placebo | | |
| --- | --- | --- | --- | --- | --- |
|  |  |  | Difference vs. placebo | 95% CI | *P* value |
| **LS mean (SE) % change in non-HDL-C from baseline** | | | | | |
| Week 12 | –41.4 (3.0) | –6.9 (4.3) | –34.5 (5.2) | –44.8 to  –24.1 | <0.0001* |
| Week 24 | –41.9 (3.1) | –6.2 (4.3) | –35.8 (5.3) | –46.3 to  –25.3 | <0.0001* |
| Week 52 | –37.5 (3.8) | –2.3 (5.3) | –35.2 (6.5) | –48.2 to  –22.2 | <0.0001 |
| Week 78 | –32.9 (4.0) | 2.9 (5.8) | –35.8 (7.1) | –49.9 to  –21.8 | <0.0001 |

The **P** value is followed by a '*' if statistically significant according to the fixed hierarchical approach used to ensure a strong control of the overall type-I error rate at the 0.05 level

CI, confidence interval; LDL-C, low-density lipoprotein cholesterol; LS, least squares; Q2W, every 2 weeks; SD, standard deviation; SE, standard error

1. **ApoB**

| All patients on background of maximally tolerated statin ± other lipid-lowering therapy | Alirocumab  150 mg Q2W (*n*=71) | Placebo (*n*=35) | Alirocumab vs. placebo | | |
| --- | --- | --- | --- | --- | --- |
|  |  |  | Difference vs. placebo | 95% CI | *P* value |
| **LS mean (SE) % change in ApoB from baseline** | | | | | |
| Week 12 | –39.2 (2.6) | –9.0 (3.7) | –30.2 (4.6) | –39.2 to  –21.1 | <0.0001* |
| Week 24 | –39.0 (2.7) | –8.7 (3.8) | –30.3 (4.7) | –39.7 to  –20.9 | <0.0001* |
| Week 52 | –35.2 (3.2) | –1.3 (4.6) | –33.9 (5.6) | –45.2 to  –22.7 | <0.0001 |
| Week 78 | –28.2 (3.5) | 6.7 (5.1) | –34.9 (6.2) | –47.2 to  –22.5 | <0.0001 |

The *P* value is followed by a '*' if statistically significant according to the fixed hierarchical approach used to ensure a strong control of the overall type-I error rate at the 0.05 level

CI, confidence interval; LDL-C, low-density lipoprotein cholesterol; LS, least squares; Q2W, every 2 weeks; SD, standard deviation; SE, standard error

1. **Total cholesterol**

| All patients on background of maximally tolerated statin ± other lipid-lowering therapy | Alirocumab  150 mg Q2W (*n*=71) | Placebo (*n*=35) | Alirocumab vs. placebo | | |
| --- | --- | --- | --- | --- | --- |
|  |  |  | Difference vs placebo | 95% CI | *P* value |
| **LS mean (SE) % change in total cholesterol from baseline** | | | | | |
| Week 12 | –33.0 (2.4) | –5.2 (3.5) | –27.8 (4.2) | –36.2 to  –19.4 | <0.0001* |
| Week 24 | –33.2 (2.6) | –4.8 (3.6) | –28.4 (4.4) | –37.3 to  –19.6 | <0.0001* |
| Week 52 | –29.7 (3.2) | –3.1 (4.4) | –26.7 (5.5) | –37.5 to  –15.8 | <0.0001 |
| Week 78 | –26.1 (3.2) | 1.4 (4.6) | –27.5 (5.6) | –38.6 to  –16.4 | <0.0001 |

The *P* value is followed by a '*' if statistically significant according to the fixed hierarchical approach used to ensure a strong control of the overall type-I error rate at the 0.05 level

CI, confidence interval; LDL-C, low-density lipoprotein cholesterol; LS, least squares; Q2W, every 2 weeks; SD, standard deviation; SE, standard error

1. **Lp(a)**

| All patients on background of maximally tolerated statin ± other lipid-lowering therapy | Alirocumab  150 mg Q2W (*n*=71) | Placebo (*n*=35) | Alirocumab vs. placebo | | |
| --- | --- | --- | --- | --- | --- |
|  |  |  | Difference vs placebo | 95% CI | *P* value |
| **Combined estimate for adjusted mean (SE) % change in Lp(a) from baseline** | | | | | |
| Week 12 | –23.2 (3.6) | –1.5 (5.1) | –21.7 (6.3) | –33.9 to  –9.4 | 0.0005 |
| Week 24 | –23.5 (3.7) | –8.7 (5.0) | –14.8 (6.2) | –26.9 to  –2.7 | 0.0164* |
| Week 52 | –33.3 (3.5) | –5.6 (5.1) | –27.7 (6.2) | –39.8 to  –15.6 | <0.0001 |
| Week 78 | –23.7 (3.7) | –1.6 (5.5) | –22.1 (6.7) | –35.2 to  –8.9 | 0.001 |

The *P* value is followed by a '*' if statistically significant according to the fixed hierarchical approach used to ensure a strong control of the overall type-I error rate at the 0.05 level

Combined estimates and standard errors (SE) are obtained by combining adjusted means and SE from robust regression model analyses of the different imputed data sets. The robust regression models include the fixed categorical effect of treatment group and randomization strata and the continuous fixed covariate of baseline Lp(a) value.

CI, confidence interval; LDL-C, low-density lipoprotein cholesterol; LS, least squares; Q2W, every 2 weeks; SD, standard deviation; SE, standard error

1. **HDL-C**

| All patients on background of maximally tolerated statin ± other lipid-lowering therapy | Alirocumab  150 mg Q2W (*n*=71) | Placebo (*n*=35) | Alirocumab vs. placebo | | |
| --- | --- | --- | --- | --- | --- |
|  |  |  | Difference vs. placebo | 95% CI | *P* value |
| **LS mean (SE) % change in calculated LDL-C from baseline** | | | | | |
| Week 12 | 7.9 (2.4) | 8.0 (3.4) | –0.1 (4.2) | –8.4 to 8.1 | 0.9727 |
| Week 24 | 7.5 (1.9) | 3.9 (2.7) | 3.7 (3.3) | –2.9 to 10.2 | 0.2745 |
| Week 52 | 7.4 (2.3) | –2.6 (3.3) | 10.0 (4.1) | 1.8 to 18.2 | 0.0175 |
| Week 78 | 8.0 (2.0) | –0.5 (2.9) | 8.5 (3.6) | 1.3 to 15.7 | 0.0210 |

CI, confidence interval; LDL-C, low-density lipoprotein cholesterol; LS, least squares; Q2W, every 2 weeks; SD, standard deviation; SE, standard error

1. **TGs**

| All patients on background of maximally tolerated statin ± other lipid-lowering therapy | Alirocumab  150 mg Q2W (*n*=71) | Placebo (*n*=35) | Alirocumab vs. placebo | | |
| --- | --- | --- | --- | --- | --- |
|  |  |  | Difference vs. placebo | 95% CI | *P* value |
| **LS mean (SE) % change in calculated LDL-C from baseline** | | | | | |
| Week 12 | –9.4 (3.7) | -4.4 (5.1) | –5.1 (6.3) | –17.3 to 7.2 | 0.4195 |
| Week 24 | –10.5 (3.3) | –1.9 (4.8) | –8.7 (5.9) | –20.2 to 2.8 | 0.1386 |
| Week 52 | –2.6 (4.8) | 4.1 (6.8) | –6.7 (8.3) | –23.0 to 9.7 | 0.4243 |
| Week 78 | –3.7 (4.5) | 8.8 (6.6) | –12.6 (7.9) | –28.1 to 2.9 | 0.1117 |

Combined estimates and standard errors (SE) are obtained by combining adjusted means and SE from robust regression model analyses of the different imputed data sets. The robust regression models include the fixed categorical effect of treatment group and randomization strata and the continuous fixed covariate of baseline fasting triglycerides value.

Apo, apolipoprotein; CI, confidence interval; HDL-C, high-density lipoprotein cholesterol; ITT, intent-to-treat; LDL-C, low-density lipoprotein cholesterol; Lp(a), lipoprotein (a); LS, least squares; Q2W, every 2 weeks; SE, standard error; TGs, triglycerides.

# Supplementary Table 5 TEAEs^a^ occurring in ≥2 % patients (safety population)

| All patients on background of maximally tolerated statin ± other lipid-lowering therapy | Alirocumab  150 mg Q2W (*n*=72) | Placebo (*n*=35) |
| --- | --- | --- |
| TEAEs occurring in ≥2 % patients in either group, % (n) |  |  |
| Infections and infestations | 43.1 (31) | 37.1 (13) |
| Nasopharyngitis | 13.9 (10) | 11.4 (4) |
| Influenza | 12.5 (9) | 2.9 (1) |
| Bronchitis | 6.9 (5) | 5.7 (2) |
| Urinary tract infection | 6.9 (5) | 0 |
| Sinusitis | 5.6 (4) | 5.7 (2) |
| Upper respiratory tract infection | 5.6 (4) | 0 |
| Laryngitis | 1.4 (1) | 2.9 (1) |
| Vulvovaginal candidiasis | 1.4 (1) | 2.9 (1) |
| Acute sinusitis | 0 | 2.9 (1) |
| Conjunctivitis | 0 | 2.9 (1) |
| Infected dermal cyst | 0 | 2.9 (1) |
| Respiratory tract infection | 0 | 2.9 (1) |
| Viral rash | 0 | 2.9 (1) |
| Neoplasms benign, malignant and unspecified (including cysts and polyps) | 0 | 2.9 (1) |
| Benign neoplasm of thyroid gland | 0 | 2.9 (1) |
| Immune system disorders | 0 | 2.9 (1) |
| Anaphylactic reaction | 0 | 2.9 (1) |
| Metabolism and nutrition disorders | 4.2 (3) | 2.9 (1) |
| Type II diabetes | 1.4 (1) | 2.9 (1) |
| Psychiatric disorders | 1.4 (1) | 8.6 (3) |
| Insomnia | 1.4 (1) | 2.9 (1) |
| Alcohol abuse | 0 | 2.9 (1) |
| Anxiety | 0 | 2.9 (1) |
| Nervous system disorders | 15.3 (11) | 8.6 (3) |
| Dizziness | 6.9 (5) | 0 |
| Headache | 6.9 (5) | 0 |
| Paresthesia | 1.4 (1) | 2.9 (1) |
| Sciatica | 1.4 (1) | 2.9 (1) |
| Amnesia | 0 | 2.9 (1) |
| Eye disorders | 4.2 (3) | 2.9 (1) |
| Iridocyclitis | 0 | 2.9 (1) |
| Ear and labyrinth disorders | 2.8 (2) | 5.7 (2) |
| Vertigo | 1.4 (1) | 5.7 (2) |
| Cardiac disorders | 12.5 (9) | 2.9 (1) |
| Myocardial infarction | 2.8 (2) | 0 |
| Angina pectoris | 1.4 (1) | 2.9 (1) |
| Angina unstable | 0 | 2.9 (1) |
| Vascular disorders | 6.9 (5) | 8.6 (3) |
| Hypertension | 4.2 (3) | 2.9 (1) |
| Hypotension | 1.4 (1) | 2.9 (1) |
| Aortic arteriosclerosis | 0 | 2.9 (1) |
| Arterial spasm | 0 | 2.9 (1) |
| Respiratory, thoracic, and mediastinal disorders | 6.9 (5) | 2.9 (1) |
| Cough | 1.4 (1) | 2.9 (1) |
| Gastrointestinal disorders | 20.8 (15) | 22.9 (8) |
| Diarrhea | 5.6 (4) | 8.6 (3) |
| Vomiting | 5.6 (4) | 0 |
| Abdominal discomfort | 2.8 (2) | 0 |
| Abdominal pain | 2.8 (2) | 0 |
| Gastro-oesophageal reflux disease | 2.8 (2) | 2.9 (1) |
| Nausea | 2.8 (2) | 5.7 (2) |
| Abdominal pain upper | 0 | 2.9 (1) |
| Dyspepsia | 0 | 5.7 (2) |
| Hemorrhoids | 0 | 2.9 (1) |
| Hepatobiliary disorders | 4.2 (3) | 2.9 (1) |
| Biliary colic | 0 | 2.9 (1) |
| Hepatic steatosis | 0 | 2.9 (1) |
| Skin and subcutaneous tissue disorders | 9.7 (7) | 2.9 (1) |
| Pruritus | 4.2 (3) | 0 |
| Systemic lupus erythematosus rash | 0 | 2.9 (1) |
| Musculoskeletal and connective tissue disorders | 20.8 (15) | 28.6 (10) |
| Myalgia | 5.6 (4) | 8.6 (3) |
| Back pain | 4.2 (3) | 0 |
| Intervertebral disc protrusion | 2.8 (2) | 0 |
| Musculoskeletal pain | 2.8 (2) | 2.9 (1) |
| Musculoskeletal stiffness | 2.8 (2) | 0 |
| Neck pain | 2.8 (2) | 0 |
| Muscle fatigue | 1.4 (1) | 2.9 (1) |
| Pain in extremity | 1.4 (1) | 2.9 (1) |
| Gouty arthritis | 0 | 2.9 (1) |
| Muscle spasms | 0 | 2.9 (1) |
| Rheumatoid arthritis | 0 | 5.7 (2) |
| Renal and urinary disorders | 4.2 (3) | 2.9 (1) |
| Renal colic | 0 | 2.9 (1) |
| Reproductive system and breast disorders | 5.6 (4) | 5.7 (2) |
| Menopausal symptoms | 0 | 2.9 (1) |
| Uterine prolapse | 0 | 2.9 (1) |
| General disorders and administration site conditions | 22.2 (16) | 8.6 (3) |
| Injection-site reaction | 8.3 (6) | 5.7 (2) |
| Fatigue | 6.9 (5) | 0 |
| Influenza-like illness | 2.8 (2) | 0 |
| Odema peripheral | 2.8 (2) | 0 |
| Pyrexia | 2.8 (2) | 2.9 (1) |
| Asthenia | 0 | 2.9 (1) |
| Pain | 0 | 2.9 (1) |
| Investigations | 15.3 (11) | 22.9 (8) |
| C-reactive protein increased | 4.2 (3) | 0 |
| Alanine aminotransferase increased | 2.8 (2) | 2.9 (1) |
| Blood creatine phosphokinase increased | 2.8 (2) | 2.9 (1) |
| Hemoglobin decreased | 2.8 (2) | 0 |
| Gamma-glutamyltransferase increased | 1.4 (1) | 2.9 (1) |
| Weight increased | 1.4 (1) | 2.9 (1) |
| Blood bilirubin unconjugated increased | 0 | 2.9 (1) |
| Blood creatinine increased | 0 | 2.9 (1) |
| Blood uric acid increased | 0 | 5.7 (2) |
| Heart rate decreased | 0 | 2.9 (1) |
| Injury, poisoning, and procedural complications | 13.9 (10) | 17.1 (6) |
| Arthropod bite | 4.2 (3) | 0 |
| Contusion | 2.8 (2) | 2.9 (1) |
| Muscle strain | 1.4 (1) | 2.9 (1) |
| Road traffic accident | 1.4 (1) | 2.9 (1) |
| Traumatic hematoma | 1.4 (1) | 2.9 (1) |
| Animal bite | 0 | 2.9 (1) |
| Joint injury | 0 | 2.9 (1) |
| Scratch | 0 | 2.9 (1) |
| Skin abrasion | 0 | 2.9 (1) |

^a^TEAEs are AEs that developed or worsened or became serious during the TEAE period (time from first dose of double-blind treatment to last dose +70 days or first dose of open-label treatment, whichever came first). More than one TEAE may be reported per patient.

AE, adverse event; Q2W, every 2 weeks; TEAE, treatment-emergent adverse event.

# Supplementary Fig 1 ODYSSEY HIGH FH study design


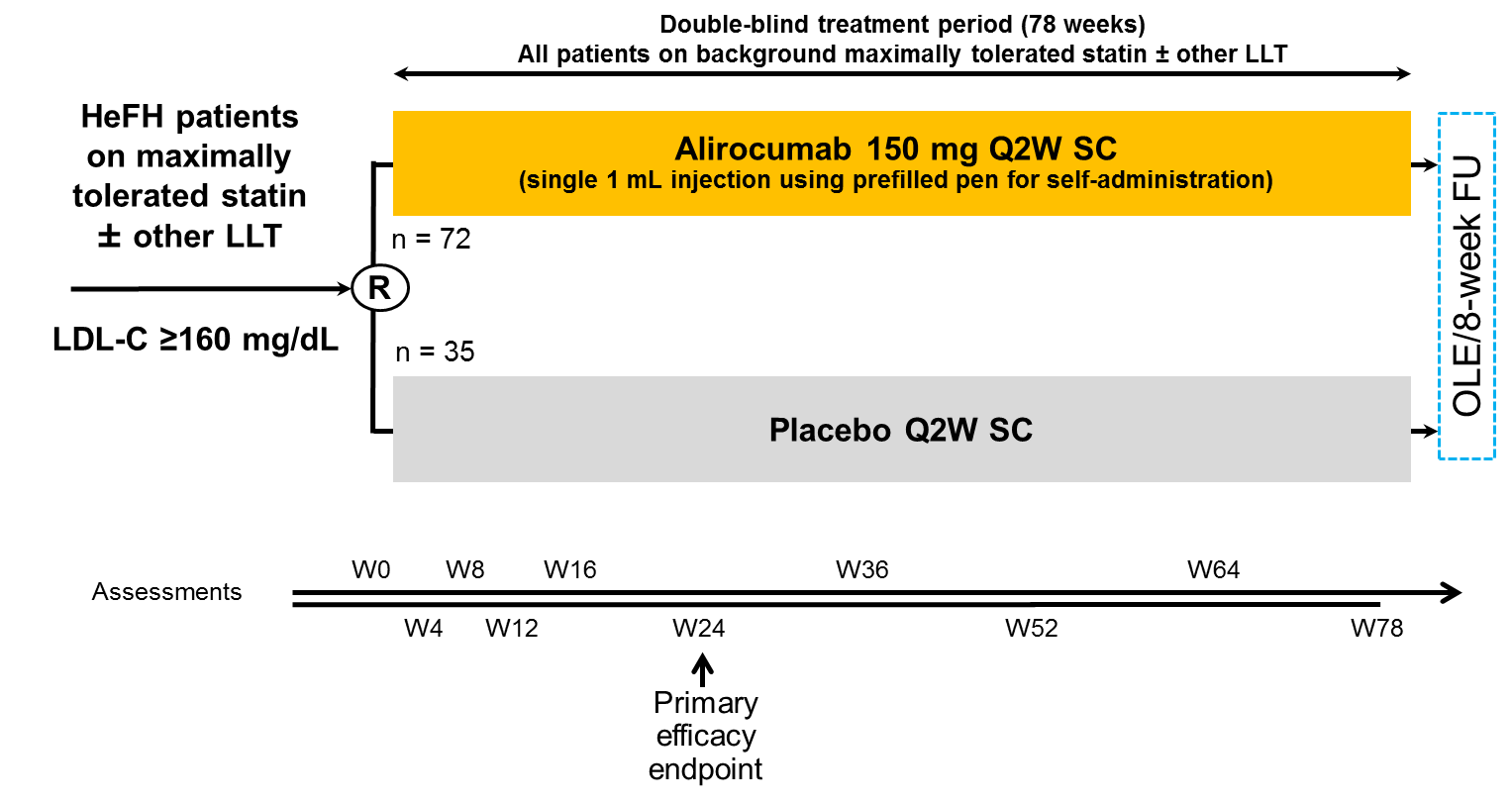


FU, follow-up; heFH, heterozygous familial hypercholesterolemia; LDL-C, low-density lipoprotein cholesterol; LLT, lipid-lowering therapy; OLE, open-label extension; Q2W, every 2 weeks; SC, subcutaneously; W, week.
